# Taxonomic colouring of phylogenetic trees of protein sequences

**DOI:** 10.1186/1471-2105-7-79

**Published:** 2006-02-17

**Authors:** Gareth Palidwor, Emmanuel G Reynaud, Miguel A Andrade-Navarro

**Affiliations:** 1 Ontario Genomics Innovation Centre, Ottawa Health Research Institute, 501 Smyth, Ottawa, ON K1H 8L6, Canada; 2 Cell Biology and Biophysics Programme, European Molecular Biology Laboratory (EMBL), Meyerhofstrasse 1, 69117 Heidelberg, Germany; 3 Department of Cellular and molecular Medicine, Faculty of Medicine, University of Ottawa, Canada

## Abstract

**Background:**

Phylogenetic analyses of protein families are used to define the evolutionary relationships between homologous proteins. The interpretation of protein-sequence phylogenetic trees requires the examination of the taxonomic properties of the species associated to those sequences. However, there is no online tool to facilitate this interpretation, for example, by automatically attaching taxonomic information to the nodes of a tree, or by interactively colouring the branches of a tree according to any combination of taxonomic divisions. This is especially problematic if the tree contains on the order of hundreds of sequences, which, given the accelerated increase in the size of the protein sequence databases, is a situation that is becoming common.

**Results:**

We have developed PhyloView, a web based tool for colouring phylogenetic trees upon arbitrary taxonomic properties of the species represented in a protein sequence phylogenetic tree. Provided that the tree contains SwissProt, SpTrembl, or GenBank protein identifiers, the tool retrieves the taxonomic information from the corresponding database. A colour picker displays a summary of the findings and allows the user to associate colours to the leaves of the tree according to any number of taxonomic partitions. Then, the colours are propagated to the branches of the tree.

**Conclusion:**

PhyloView can be used at . A tutorial, the software with documentation, and GPL licensed source code, can be accessed at the same web address.

## Background

Phylogenetic trees based upon multiple sequence alignments of proteins from many species are commonly used to determine the evolutionary relationships between homologous sequences, which can give insights into the evolution of a protein family and the functional specificity of the members of the family [1].

It is expected that the phylogenetic tree reflects the events of duplication and speciation of proteins. Through speciation, related proteins in different organisms are generated that reflect the taxonomic relations of those organisms. However, cases of phylogenetic associations being at odds with known taxonomy can be interesting anomalies worthy of investigation, perhaps indicating problems in the generation of the multiple sequence alignment or events of lateral gene transfer [2]. Also, events of gene duplication (that may define subfamilies with subtle variations of the common functional theme of the family) can be observed by the repetition of a taxonomic structure at multiple places in the tree.

Unfortunately, available software for rendering phylogenetic trees does not provide a simple means of automatically retrieving taxonomic information for the sequences represented in the tree, or of graphically representing arbitrary taxonomic properties of trees, thus allowing the study of the relation of a phylogenetic tree with the taxonomic relations between the species represented in the tree. PhyloView was developed in an attempt to address this limitation and provide a simple and generic means of doing this.

## Implementation

PhyloView was written as a web application in Perl 5.6.1 using the Perl modules BioPerl [3], Parse::RecDescent, SVG and CGI. Full source code for the program is available at the PhyloView web site [4], has been deposited at SourceForge.net [5] and is licensed under the GPL [6].

## Results and discussion

Upon initial loading the script provides a form where the user may upload a phylogenetic tree in Newick (New Hampshire) format, used by most phylogenetic packages (e.g., [7]). The tree should contain SwissProt, SpTrembl [8], or GenBank GI protein identifiers [9] in the leaf node names. The associated records are then dynamically retrieved from the appropriate database online over the internet and the associated taxonomic information is extracted. Processing times for the initial upload of a tree vary with the number of sequences in the tree, the load on the server and the response speed of the public databases queried. For example, a tree with 600 sequences (which in our experience is pretty large for a phylogenetic tree) takes about one minute to load. Subsequent processing times for the same tree tend to be much shorter as the taxonomic information associated with the protein sequence identifiers is cached and no further queries of public databases are required.

To allow users to show their own identifiers in the tree, we use the following internal format for names: "DBID*YOURID" where DBID is the database identifier used by PhyloView to extract the taxonomic information, YOURID is the identifier to be displayed in the tree, and * is a user defined separator (the default symbol is "/"). Optionally, the DBID can be removed from the rendered tree leaving the user identifiers.

**Figure 1:**
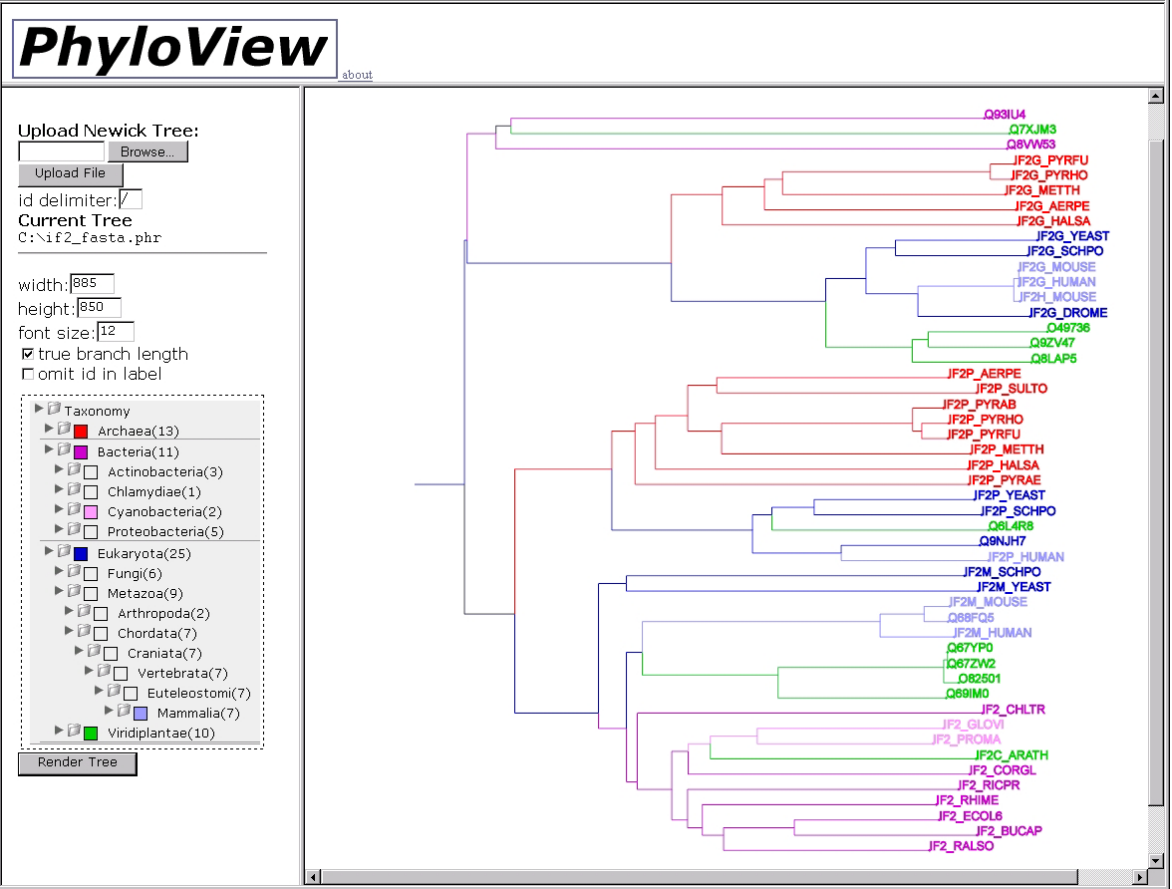
**PhyloView web interface**. The phylogenetic tree is input on the top left window. Bottom-left: summary of the taxonomic levels present in the tree (with number of sequences in each within brackets) that can be expanded and contracted at will. A colour picker allows the association of a colour with any taxonomic level. Right: interpretation of a tree. In this example, a multiple sequence alignment of putative transcription initiation factor 2, gamma subunit, and related sequences, is used to illustrate PhyloView (the example is available at the web site). Colouring chosen is: Archaea:red; Bacteria:pink; Cyanobacteria:light pink; Eukarya:blue; Viridiplantae:green; Mammals:light blue. Repeating phylogenetic structures make obvious the existence of two subfamilies (IF2G, and a hypothetical IF2P), and the presence of three outliers (top: three GTPases of unknown function, wrongly included in the alignment). The plant sequence that groups with the Cyanobacteria (IF2C_ARATH) is a chloroplast IF2G. The eukaryotic members that group with bacteria (IF2M) are mitochondrial IF2Gs. Recent duplications of mammalian IF2Gs are also apparent.

The provided Newick tree is parsed by the Perl RecDescent module. The protein IDs of the Newick tree leaves are extracted and the associated taxonomic identities are extracted from the SwissProt, SpTrembl, and GenBank databases. A taxonomic tree is then generated containing the aggregate taxonomic information of the tree leaves and represented on the resulting web page as clickable tree menu with a JavaScript colour picker at every node (see Figure 1). This mechanism allows the user to associate colours to arbitrary taxonomic groups, with the initial defaults being Eukarya:blue, Bacteria:green, Archaea:red, and unknown:black.

Once the colours have been chosen, resubmitting the form will render a new tree where the various nodes and branches are coloured based upon the above choice. The taxonomic colouring algorithm is such that every branch of the tree receives the colour assigned to the taxonomic group with most members under that branch. In case of a tie between assignments, the more specific one is given precedence (for example, Viridiplantae over Eukarya). Colouring of a given branch only happens if more than 50% of the sequences under that branch belong to a single taxonomic group with an assigned colour.

Mouse-over of the phylogenetic tree leaf nodes in SVG mode creates floating tool-tip type output with full taxonomic information for the sequence. The preferred form of output for the tree is an SVG image. SVG is an XML based standard for vector graphics. Though not natively supported by most browsers, a number of plug-ins is freely available, for example the Adobe SVG viewer [10].

We plan to extend PhyloView as a visualization framework for enhancing sequence phylogenetic tree images with associated data. We welcome feedback and proposals for additional features from users.

## Conclusion

PhyloView is the first web server dedicated to colouring according to taxonomy of phylogenetic trees. There is other software that may be used to attain similar results but with considerably more effort. For example, Mesquite [11] (an open source modular software system for evolutionary analysis written in Java) and MacClade [12] (a commercial computer program for phylogenetic analysis that runs only on MacOS), allow the manual colouring of the branches of a phylogenetic tree, but these are complicated general purpose programs and achieving this is a laborious and complicated process. PhyloView is intended to streamline and simplify this, allowing the user to rapidly explore different combinations of colours and taxonomic partitions for the best visual result.

## Availability and requirements

PhyloView requires Internet Explorer with the Adobe SVG viewer plug-in and can be used at [4]. Source code is available from that location as well.

## Authors' contributions

MA and ER conceived the tool. GP implemented the tool. GP and MA drafted the manuscript. All authors tested the tool during its development, and read and approved the final manuscript. MA was previously known as Miguel A. Andrade.
